# Beyond Traditional Sunscreens: A Review of Liposomal-Based Systems for Photoprotection

**DOI:** 10.3390/pharmaceutics16050661

**Published:** 2024-05-15

**Authors:** Júlio Abreu Miranda, Yasmin Ferreira da Cruz, Ícaro Chaves Girão, Fabia Julliana Jorge de Souza, Wógenes Nunes de Oliveira, Éverton do Nascimento Alencar, Lucas Amaral-Machado, Eryvaldo Sócrates Tabosa do Egito

**Affiliations:** 1 Graduate Program in Health Sciences, Federal University of Rio Grande do Norte (UFRN), Natal 59012-570, Brazil; julioabreumiranda@gmail.com (J.A.M.); fabiajulliana@gmail.com (F.J.J.d.S.); wogenes95@gmail.com (W.N.d.O.); socratesegito@gmail.com (E.S.T.d.E.); 2 Pharmacy Department, Federal University of Rio Grande do Norte (UFRN), Natal 59012-570, Brazil; yasmin.fereira.11@ufrn.edu.br (Y.F.d.C.); chavesicaro15@gmail.com (Í.C.G.); 3 Laboratory of Micro and Nanostructured Systems (LaSMiNano), College of Pharmaceutical Sciences, Food and Nutrition, Federal University of Mato Grosso do Sul (UFMS), Campo Grande 79070-900, Brazil; everton_alencar@hotmail.com; 4 School of Pharmaceutical Sciences, São Paulo State University (UNESP), Araraquara 14800-903, Brazil

**Keywords:** liposomes, ultraviolet radiation, solar damage, photoprotection, vesicular systems

## Abstract

Sunscreen products are essential for shielding the skin from ultraviolet (UV) radiation, a leading cause of skin cancer. While existing products serve this purpose, there is a growing need to enhance their efficacy while minimizing potential systemic absorption of UV filters and associated toxicological risks. Liposomal-based formulations have emerged as a promising approach to address these challenges and develop advanced photoprotective products. These vesicular systems offer versatility in carrying both hydrophilic and lipophilic UV filters, enabling the creation of broad-spectrum sunscreens. Moreover, their composition based on phospholipids, resembling that of the stratum corneum, facilitates adherence to the skin’s surface layers, thereby improving photoprotective efficacy. The research discussed in this review underscores the significant advantages of liposomes in photoprotection, including their ability to limit the systemic absorption of UV filters, enhance formulation stability, and augment photoprotective effects. However, despite these benefits, there remains a notable gap between the potential of liposomal systems and their utilization in sunscreen development. Consequently, this review emphasizes the importance of leveraging liposomes and related vesicular systems as innovative tools for crafting novel and more efficient photoprotective formulations.

## 1. Introduction

Ultraviolet (UV) radiation is one of the main contributing factors for skin damage due to its ability to cross the ozone layer [1]. Sunburn, skin elasticity reduction, wrinkle formation related to premature aging, and cellular DNA damage (a determining factor for skin carcinogenesis) are the main damages caused by UV rays [2]. Thus, skin protection against this harmful radiation is mandatory to preserve skin health and avoid skin cancer. This protection can be achieved by using physical barriers, such as sunglasses, umbrellas, and hats, combining these with photoprotective cosmetic formulations, popularly named sunscreens [3].

Photoprotective formulations are characterized by UV filters, which are classified as organic and inorganic according to their chemical structure and mechanism of action [4]. Additionally, several photoprotective formulations are currently available on the market as emulsions for topical application [5]. Although current emulsions can deliver UV filters and assure client compliance, their physical instability may lead to phase separation, and the chemical instability of UV filters in this system may compromise the product’s efficacy [6]. Despite the low water resistance commonly found in such formulations, this parameter is fundamental for sunscreens when used during recreational activities, like swimming [7]. Indeed, water resistance is often limited by the oil/water/surfactants composition of the traditional emulsions [6].

Furthermore, the choice of sunscreen filter significantly influences user compliance. Inorganic filters, commonly employed in sunscreens, can sometimes leave a white residue on the skin, potentially reducing user acceptance [8]. Consequently, an optimal sunscreen formulation should encompass several key attributes: (i) UV filters with robust stability, (ii) effective retention of filters within the skin’s uppermost layers, (iii) preservation of the skin’s natural appearance, (iv) water resistance, and (v) long term stability of the product [9].

In this context, numerous studies have been directed towards the development of novel products and systems to enhance the safety and effectiveness of photoprotective formulations [8]. Among these, nanosystems, including nanoemulsions, solid lipid nanoparticles, and polymeric nanoparticles, have emerged as promising platforms for photoprotection [10,11,12,13]. Beyond that, liposomes as vesicular systems warrant greater attention for their applicability in photoprotection [14]. While many studies highlight other nanosystems for this purpose, it is evident that liposomal-based systems should not be overlooked. Due to their unique physicochemical characteristics, liposomes can effectively encapsulate both lipophilic and hydrophilic UV filters, enabling the formulation of broad-spectrum sunscreen products [15]. Furthermore, their utilization in photoprotection can contribute to the development of more eco-friendly formulations, particularly due to their water resistance. Therefore, the objective of this narrative review is (i) to elucidate the characteristics of liposomal-based systems, (ii) to illustrate how these nanosystems contribute to the creation of new sunscreen formulations, and (iii) to provide an updated perspective on current research in liposomal-based photoprotection.

## 2. Ultraviolet Radiation and Photoprotection

The sun is a source of various forms of radiation, such as infrared, visible light, and UV radiation. This UV radiation comprises a spectrum between 10 and 400 nm, being the most interactive radiation with the skin due to its high energy and small wavelength, facilitating its permeation through the skin barrier [2]. UV light is important in natural processes, such as vitamin D production, which happens by its interaction with 7-dehydrocholesterol at the epidermis [16]. It also has a therapeutic use in the treatment of immunosuppressive patients that requires local therapy [17].

Although UV light provides benefits to the homeostasis, it has become an undeniable threat to human skin health over the decades, especially because of ultraviolet A (UVA) (320–400 nm) and ultraviolet B (UVB) (295–320 nm), which can cross the ozone layer and penetrate the skin [18,19,20]. This radiation leads to detrimental effects such as premature aging, erythema, low elasticity, DNA damage, and an increased risk of skin cancers [19,20].

UVA rays, characterize by longer wavelengths, possess the remarkable capability to penetrate deep into the dermis, the skin’s thickest layer, thereby inducing oxidative stress and accelerating photoaging [21]. Conversely, UVB rays, while not penetrating the skin as deeply as UVA rays, primarily contribute to sunburn and can instigate the onset of skin cancer [20]. The intricate interaction between these two types of UV light underscores the critical necessity for robust photoprotection strategies aimed at mitigating their harmful effects [22]. Photoprotection encompasses a wide array of strategies designed to minimize the adverse effects of solar radiation on the skin. Among these, photoprotective formulations, such as sunscreens, have emerged as a key strategy in combating UV-induced damage [2].

### Sunscreen Formulations

Sunscreens, cosmetics designed to shield against UV rays, typically contain UV filters [23]. These formulations are often supplemented with additional compounds, including fragrances, preservatives, stabilizers, emulsifiers, emollients, and other chemicals aimed at enhancing their sensory attributes and effectiveness [3].

These products function by either absorbing, reflecting, or scattering harmful UV rays, thereby thwarting their penetration into the deeper layers of the skin [24]. As mentioned earlier, sunscreens are predominantly available in the form of emulsions, comprising an aqueous and oily blend stabilized by surfactants and supplemented with UV filters [6]. These filters are categorized as organic or inorganic based on their chemical structure and mechanism of action [3]. Organic UV filters can absorb specific wavelengths and are classified as UVA-absorption filters, UVB-absorption filters, or broad-spectrum UV filters (capable of absorbing UVA and UVB) [25]. In contrast, inorganic UV filters typically absorb, reflect, and scatter radiation. It is also crucial to note that for effective photoprotection, the filters in the formulations must remain within the stratum corneum (SC) and should not penetrate the deeper layers of skin [26,27].

Existing sunscreen products on the market often have drawbacks, such as high opacity on the skin due to the size of particles used in inorganic UV filters like zinc oxide (ZnO) and titanium dioxide (TiO_2_) [7]. To address this issue, some manufacturers have attempted to reduce the size of these particulate filters. However, this alteration has led to a decrease in spectral absorption and increased the likelihood of the filters reaching the dermis or permeating through the skin, as evidenced by their presence in the blood and urine of volunteers [8].

In response, innovative technologies like nanotechnology have been explored to overcome these challenges [28]. Nanotechnological sunscreen formulations offer improved efficiency and stability of UV filters while reducing their bioavailability [29]. These formulations may include nanoemulsions, nanocapsules, nanoparticles, and liposomal-based systems [14,28,30]. Despite their smaller particle or droplet sizes, these systems can be engineered to prevent penetration and remain on the outer layers of the skin [31]. Achieving this characteristic can enhance filter entrapment, protection, and overall sunscreen performance.

## 3. Liposomes

Liposomes are vesicular systems composed of phospholipids, sterols, and polysaccharides, organized in bilayers through lipid interactions [32,33]. Phospholipids are the primary constituents of liposomes, characterized by a chemical structure comprising hydrocarbon tails and a polar head region, facilitating interactions with both hydrophilic and hydrophobic substances [33,34]. Phosphatidylcholine, phosphatidyletanolamine, and phosphatidylglycerols are the main phospholipids used in liposome production. Among them, phosphatidylcholine is notable for its cylindrical shape, biocompatibility, and stability over a wide range of pH values and salt concentrations [33,34,35].

These vesicular systems can be categorized based on their size, the number and organization of bilayers, and their composition. This classification is crucial as the characteristics of liposome structure determine their size and impact their internalization or retention [33,36,37]. Moreover, it dictates the amount of active compound that can be incorporated and facilitates the modulation of release rate [32,36].

Similarly relevant, the phase transition of liposomes is another fundamental aspect of these structures. Phase transition affects the integrity and behavior of phospholipids according to temperature variations [32]. It indicates changes in the physical state of the lipid bilayer, which can transition from an ordered gel-shaped hydrocarbon chain conformation to a disordered liquid-crystalline phase in response to temperature changes [38]. This transition is influenced by factors such as the length and saturation of the lipid chain. Therefore, it is possible to modulate the fluidity of liposomes by selecting specific phospholipids for their production. The phase transition also impacts the permeability of liposomes through tissues. Low permeability occurs when the temperature is below the phase transition of liposomes [32,39]. In the context of using liposomes as vehicles for UV filters, one must understand that the phase transition is crucial for their topical application and retention of filters on the skin surface.

Moreover, various approaches are employed to enhance the fluidity of these systems, such as the addition of ethanol or surfactants as membrane modifiers. This results in the formation of new vesicles known as ethosomes, transfersomes, and transethosomes, which will be discussed further. Ethanol and surfactants interact with the lipid components of the stratum corneum (SC), disrupting the tightly packed lipids, altering the structure of keratinized or lipophilic domains, and reducing the transition temperature of lipids [40]. Additionally, these membrane modifiers penetrate the hydrocarbon chains and alter the net charge of the vesicles, leading to a reduction in their size. Consequently, researchers have utilized these compounds as permeation enhancers [40,41].

Hence, the physicochemical properties of the discussed liposomes reinforce their potential as topical sunscreen vehicles. Their biocompatible phospholipids, coupled with their physical structure capable of encapsulating various UV filters, as well as their phase transition properties enabling filter retention on skin’s surface layers, underscore their utility in sunscreen formulations [42,43,44].

## 4. Skin and Topical Delivery of Liposomes

The skin, as the largest organ in the human body, serves as the primary barrier against external agents. It regulates the entry of microorganisms, maintains the body temperature, and controls the physiological water levels. Comprising three layers of biological tissues with distinct architecture and functionality—epidermis, dermis, and hypodermis—the skin plays a crucial role in overall health [45,46,47,48].

The epidermis, the outermost layer, consists of keratinocytes, melanocytes, Langerhans cells, and Merkel cells [49,50]. Its surface, known as stratum corneum, is composed of flattened corneocytes surrounded by a lipid matrix [49]. The dermis, the skin’s intermediate layer, contains elastin, collagen fibers, nerves, macrophages, sweat and sebaceous glands, hair follicles, and lymphatic vessels. This layer is nourished by blood vessels to provide structural support [51]. The hypodermis, the innermost layer, primarily consists of adipocytes, fibroblasts, and macrophages. It functions to protect against shock, conducting nerve signals, provide thermal insulation, and serve as the body’s energy reserve [48,52,53].

In this context, the skin tissue architecture allows for its utilization as a unique administration route for various drugs and active compounds incorporated into delivery systems, such as liposomes [54,55,56]. Liposomes typically release active compounds upon contact with skin cells; however, it is well-established that several factors influence the permeation and efficacy of liposomes in the skin. These factors include the concentration of active compounds, physicochemical characteristics, composition, and the production method of the formulation [52,56]. Additionally, skin conditions such as integrity and hydration play a crucial role [54].

While liposomes have demonstrated utility in enhancing the delivery and absorption of active compounds into the skin, it is important to note that the delivery of the encapsulated molecules in these vesicles relies on the type and composition of the liposome [54,56,57,58]. Various mechanisms have been proposed for the skin delivery of liposomes, including (i) adsorption or fusion with the SC, which is commonly observed conventional vesicles composed solely of phospholipids, and (ii) penetration through the skin pores or cutaneous appendix, which is associated more with membrane-modified liposomes such as ethosomes, transfersomes, and transethosomes, facilitating greater drug permeation through the skin [37,59,60]. While the use of conventional liposomes for drug delivery has diminished due to these mechanisms, they still hold promise as a potential system for sunscreen development [37].

This statement is supported by the study conducted by Manosroi et al. [61], in which liposomes with and without membrane modifiers were developed. Permeation studies using a rat skin model revealed that conventional liposomes were not detected in the receiving chamber, whereas the membrane-modified liposomes were able to penetrate the skin. Similarly, Verma et al. [62] investigated the in vitro permeation of conventional liposomes using a human skin model and observed that they predominantly remained within the SC, with minimal penetration into deeper skin layers and the receiver chamber. These findings underscore that conventional liposomes tend to remain in the upper layers of the SC and viable epidermis without deeply penetrating the skin. This behavior can be attributed to the interaction between liposome phospholipids and the skin’s cellular lipids, which likely maintain cutaneous integrity, facilitating release within the SC while preventing deeper permeation [37,61,62].

In pursuit of more effective and user-friendly sunscreens, the incorporation of liposomes into sunscreen formulations has emerged as an advantageous approach. Liposomes have garnered attention due to their unique ability to encapsulate both hydrophilic and lipophilic agents, thereby enhancing the stability and efficacy of sunscreen agents [15]. The integration of liposomes into sunscreens represents a significant advancement in photoprotective formulations. By encapsulating UV filters within liposomes, it is possible to achieve a more even distribution and retention of the active ingredients on the skin compared to regular sunscreens, thereby enhancing the protective barrier against UV radiation [15,63,64].

Additionally, liposomes can facilitate the controlled release of UV filters, extending the protection time and decreasing the frequency of application [65]. Furthermore, the biocompatibility and versatility of liposomes make them an ideal carrier for antioxidants and other skin-beneficial compounds. This feature provides a dual-action approach by protecting the skin against UV light and actively counteracting the oxidative stress induced by solar radiation [63,66]. This strategic use of liposomes in sunscreens elevates the standard of photoprotection and opens new pathways for developing more efficient and user-friendly sun care products.

### 4.1. Liposomes as a Strategy for Ulraviolet Filter Delivery

A suitable sunscreen formulation should allow (i) a proper incorporation of effective UV filters, (ii) chemical stability of the filters, (iii) overall product stability over time, and (iv) topical delivery of incorporated filters, which must remain on the skin surface and SC [2]. If the filters permeate through the skin, they can reach the dermis and enter the blood vessels, leading to systemic distribution and toxic effects. Furthermore, as the filters act by reflecting, absorbing, and scattering UV light, they must be placed on the skin surface to promote suitable photoprotection [67].

As previously stated, liposomes can incorporate a wide range of photoprotective ingredients [42]. Additionally, once applied to the skin’s surface, liposomes remain in the SC due to their low permeability in intact skin structure [68].

Due to the similarity between liposomes structure and skin cells, the liposomal bilayer merges with the cellular membrane’s phospholipid bilayer at the SC, where the UV filters should remain retained for photoprotective action [69,70,71]. Moreover, due to the presence of phospholipids in both liposomes and the SC, this system presents a suitable biocompatibility with the skin, reducing irritation and allergic reactions [44,64].

**Figure 1:**
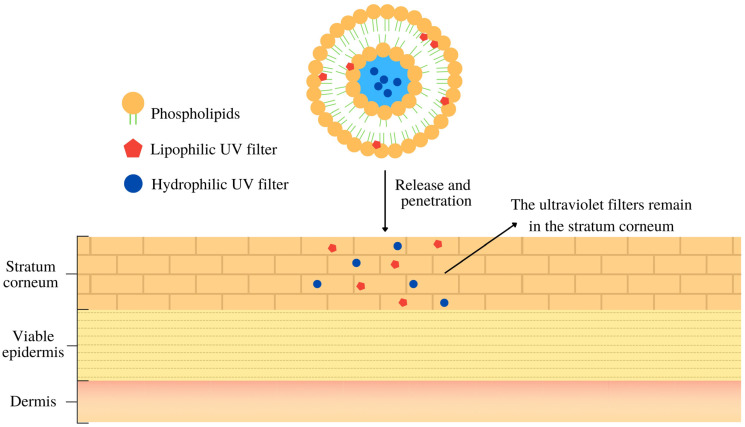
Schematic representation of a proposed action mechanism of liposome-loaded ultraviolet filters on the stratum corneum.

By utilizing liposomes, a strategy for achieving a broad spectrum of UV radiation protection can be implemented. These structures enable the incorporation of hydrophilic and lipophilic substances (Figure 1), allowing for the inclusion of organic and inorganic UV filters with different polarities within the same formulation [44,72,73]. For instance, in a hypothetical photoprotective liposome, hydrophilic UV filters, such as phenylbenzimidazol sulfonic acid and para-amino benzoic acid, would be housed in the aqueous nucleus, while lipophilic filters like butyl methoxy dibenzoylmethane (BMBM), and octyl methoxycinnamate (OMC) would be entrapped within the lipid bilayers. This approach has been adopted in several studies utilizing liposomes as nanostructured systems for UV filter delivery. Table 1 provides a summary of these studies, which is further discussed in this paper.

Various studies have explored the utilization of liposomes for delivering OMC (octyl methoxycinnamate) and BMBM (butyl methoxy dibenzoylmethane), commonly known as avobenzone. Both of these organic filters are extensively employed in photoprotective formulations due to their ability to absorb a broad spectrum of UV radiation absorption, encompassing both UVA and UVB rays, especially when combined [76]. Despite the OMC effectiveness, studies have indicated its penetration into deeper layers of the skin, including the dermis and adipose tissue, with systemic absorption evidenced by its presence in urine and milk samples following topical application [77]. Conversely, BMBM demonstrates high efficacy against the UVA radiation spectrum, yet its photostability is compromised, impacting its effectiveness in photoprotective formulations. This instability arises from tautomeric (enol and keto) forms of BMBM, where the enol form absorbs UVA radiation but undergoes photoisomerization to the keto form, which lacks UVB or UVA radiation [78,79]. Consequently, several studies have investigated liposomal formulation of OMC and BMBM to enhance the filters’ stability, skin retention, and overall efficacy [15,63,65,74].

Mota et al. incorporated OMC into liposomes and compared the formulation with free OMC regarding its photoprotective activity and skin permeation [65]. The authors observed a higher in vivo sun protection factor (SPF) value for the liposomal OMC (11.50 ± 2.70) compared to the free OMC formulation (7.00 ± 1.60). Additionally, their biodistribution study identified UV filter deposition in the liver after free OMC use, indicating systemic absorption from the skin. In contrast, the liposomal OMC formulation remained at the skin surface with minimal permeation, thus avoiding systemic absorption. Therefore, this study demonstrated the ability of liposomes to retain UV filters on the surface and enhance their efficacy.

In a separate study, conducted by Monteiro and colleagues, the delivery of OMC from different systems was compared: (i) liposomes containing OMC (lipo/OMC), (ii) OMC incorporated into β-cyclodextrin (β-CD/OMC), (iii) OMC incorporated in liposomes and β-cyclodextrin (lipo/OMC + β-CD/OMC), and (iv) free OMC [74]. The authors determined the in vivo SPF of the formulations, their water resistance, and in vitro permeation. The results showed that the lipo/OMC formulation exhibited the highest in vivo SPF (11.00 ± 1.30), which remained unaffected by the water immersion (10.30 ± 2.20). Furthermore, lipo/OMC showed the lowest in vitro permeation among all tested formulations. Thus, the findings emphasized the ability of liposomes to enhance the efficacy of UV filters; improve skin retention; and demonstrate water resistance, a crucial parameter for sunscreen formulations.

The study by Caldas et al. aimed to produce and incorporate BMBM while enhancing its photostability in various nanosystems, including liposomes, solid lipid nanoparticles, and nanostructured lipid carriers [63]. Additionally, omega-3 was added to all systems to achieve multifunctionality. The authors evaluated the photostability of BMBM after its incorporation in all lipid systems, both with and without omega-3. The results demonstrated a significant improvement in BMBM photostability for all nanosystems, suggesting that omega-3 did not affect the BMBM stability. The stability enhancement was attributed to the incorporation of BMBM into nanostructured systems, further emphasizing the ability of nanostructured delivery systems, including liposomes, to protect UV filters against chemical degradation and instability phenomena.

In a different approach, Xu et al. synthesized a new molecule derived from BMBM, namely, 4-cholesterocarbonyl-4′-(N,N′-diethylaminobutyloxy) azobenzene (CDBA), which was incorporated into liposomes [75]. This system was evaluated for CDBA’s photostability, photoprotective action, and content release. The results indicated that liposome incorporation successfully improved CDBA’s photostability, provided suitable in vitro photoprotection against UVA and UVB radiation, and achieved photo-controlled release.

Severino et al. also investigated the ability of liposomes to enhance the efficacy of UV filters [64]. They examined the UV absorption of benzophenone-3 incorporated into a liposomal formulation. The data revealed a high entrapment efficiency of benzophenone-3 in the liposomes. Although quantitative SPF results were not provided, the authors conducted a qualitative study by evaluating the absorption spectra of the developed formulation. The data revealed a high entrapment efficiency of benzophenone-3 in the liposomes, leading to increased UV absorption compared to the free UV filter solution. Moreover, liposomes improved the chemical stability of the filter, further highlighting the advantages of these vesicular systems for sunscreen applications.

These studies collectively underscore the potential of liposomes in developing new photoprotective formulations. Liposomes offer the capability to carry various UV filters, enhance their efficacy and chemical stability, prevent photounstability, retain molecules on the skin surface, improve water resistance, and prevent systemic absorption and associated side effects.

### 4.2. Other Vesicular Systems for Application in Photoprotection

Although liposomes offer numerous advantages for delivering UV filters and advancing sunscreen formulations, they are susceptible to a drawback that can compromise its effectiveness over time: the gradual release of incorporated molecules during storage. Liposomes are prone to hydrolysis in aqueous media, leading to the disruption of phospholipid bilayers and gradual release of the active compounds under storage conditions, which can compromise product stability and effectiveness [31]. To overcome this limitation, researchers have proposed the use of liposomes combined with biodegradable polymers to generate hybrid liposomes, presenting a smart strategy [31].

Pavelkova et al. developed hybrid liposomal vesicles containing poly (3-hydroxybutyrate) (PHB) for the delivery of coffee extracts (*Coffea arabica*), aiming to enhance long-term product stability [31]. Herein, the authors did not used a synthetic ultraviolet filter but a natural product rich in phenolic compounds responsible for the photoprotective activity. The study utilized natural coffee extracts rich in phenolic compounds, known for their photoprotective activity and multifunctional properties such as antioxidant, skin-regenerative, and moisturizing effects [31,63,66,80]. Results demonstrated that green and roasted coffee extracts provided antioxidant activity and high values of SPF in vitro, which is around 40 to 50, according to the total phenolic content of each type of extract.

Under storage conditions, the extract alone presented a decrease in SPF of around 50% under one hour, whereas the PHB-liposomes showed a decrease in approximately 30% after two months. Moreover, the results showed that incorporating PHB into liposomes improved stability, with PHB-liposomes exhibiting higher SPF stability compared to crude coffee extracts and liposomes without the polymer. Physicochemical characteristics such as zeta potential, particle size, and polydispersity index (PdI) showed no significant changes, demonstrating the enhanced stability conferred by PHB. Conversely, liposomes without PHB showed significant variations in such parameters. This study demonstrated that adding PHB to a liposome, compared to a traditional liposome, provides suitable chemical stability of the phenolic compounds and appropriate physicochemical stability of the vesicles to the formulation.

Similarly, Castro et al. developed hybrid liposomes containing chitosan for delivering OMC, demonstrating suitable stability and increased SPF compared to free OMC formulations [15]. Additionally, the formulations showed a lower concentration and encapsulation of UV filter. Also, the value of SPF of the formulation was larger than the ones revealed using the in silico method. Chitosan-coated liposomes exhibited slower release kinetics compared to formulations without chitosan, indicating the potential of polymeric particles in enhancing UV radiation reflection and synergizing with UV filters.

**Figure 2:**
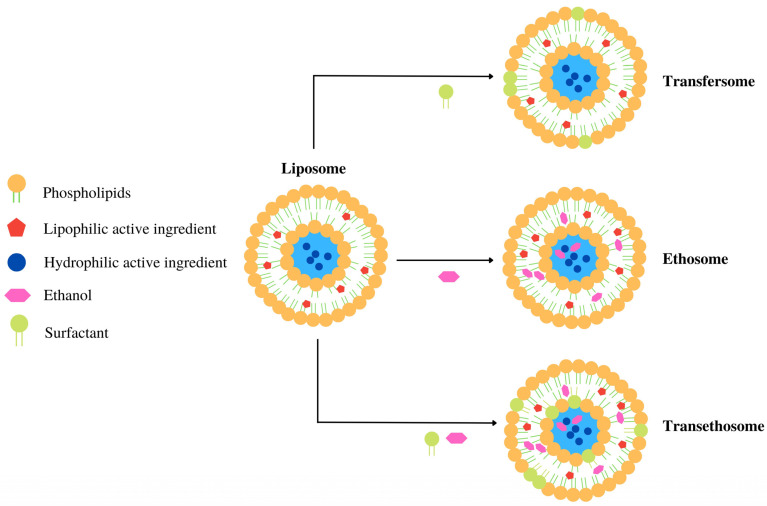
Structural representation of liposomes and their derivatives, including transfersomes, ethosomes, and transethosomes. These variations are developed by adding a surfactant, an ethanol, and a mixture of surfactant/ethanol to liposomes.

The incorporation of polymers to produce liposomes offers a promising avenue for developing new sunscreen formulations with enhanced long-term stability and improved efficacy and safety. Beyond the use of polymers to improve the characteristics of liposomes, new vesicular systems have been developed using these traditional systems as templates. Indeed, innovative vesicular systems derived from liposomes, such as transfersomes, ethosomes, and transethosomes, have been developed to address liposomes’ limitations by enhancing permeation to deeper skin layers [60]. Transfersomes possess surfactants attached to the liposome’s lipid bilayer, while ethosomes present alcohol molecules in the lipid bilayer. Finally, transethosomes are produced by adding ethanol and surfactants during the vesicle formation [59]. These liposomal derivatives, obtained by adding or replacing chemical compounds, represent a significant advancement in sunscreen delivery systems, as depicted in Figure 2.

As mentioned previously, the retention of UV filters in the stratum corneum (SC) is essential for the effectiveness of formulations against UV radiation. This requirement could potentially limit the use of these liposomes derivatives for this purpose [44]. However, these derivatives are valuable for delivering of other compounds in multifunctional formulations. This innovative approach has been extensively employed in the cosmetic industry to create sunscreens with antioxidant, skin-regenerating, anti-aging, or moisturizing properties, thereby ensuring more comprehensive skin care and protection [66,81]. Consequently, the production of liposome derivatives has the potential to enhance the development of multifunctional photoprotective formulations, which not only contribute to shielding the skin against ultraviolet radiation but also promote skin health through various mechanisms.

To develop a multifunctional formulation with photoprotective, antioxidant, and anti-aging properties, Gollavilli et al. incorporated naringin, a flavonoid found in grapefruit, into ethosomes. These vesicles were then integrated into photoprotective creams containing dispersed nanoparticles of zinc oxide and titanium dioxide [66]. Interestingly, the presence of naringin ethosomes did not impact the SPF of the final formulation. Formulations with naringin ethosomes alone exhibited an SPF of 1.16 ± 0.09, whereas formulations with both naringin ethosomes and UV filters displayed an SPF of 21.21 ± 0.62. This SPF value was solely attributed to the synthetic inorganic filters.

Although naringin did not contribute to the SPF of the formulation, it significantly enhanced the antioxidant activity of the final product. In vitro assessments of naringin ethosomes revealed an outstanding free radical scavenging activity. The authors further investigated the skin permeation of the creams containing naringin ethosomes and UV filters, observing that naringin penetrates deep into the skin layers, while the filters remained on the superficial layers. This approach suggests that despite their increased permeability, ethosomes, transfersomes, and transethosomes could be combined for the development of multifunctional sunscreens.

## 5. Final Considerations

Sunscreens are essential tools for protecting the skin against UV radiation. Consequently, there is a growing demand in the market for new products that offer improved efficacy, safety, chemical stability, and multifunctional properties. As a result, researchers worldwide are increasingly focused on developing new sunscreen formulations. Liposomes and their derivatives present a promising alternative for the future of the cosmetic sunscreen industry, as they can address these challenges. Liposomes offer several advantages for sunscreen formulations. They enhance the efficacy of UV filters, ensuring the retention on human stratum corneum to provide effective photoprotection while ensuring safety. Additionally, liposomes improve the chemical stability of UV filters and reduce photounstability phenomena, thereby enhancing overall product biocompatibility.

Moreover, hybrid liposomes and liposome derivatives, such as ethosomes, transfersomes, and transethosomes, contribute to the development of multifunctional formulations with additional antioxidant, anti-aging, skin-regenerating, and moisturizing activities. This approach supports the maintenance of healthy skin characteristics by directly addressing photoprotection and repairing skin damage from various sources.

Despite the numerous advantages and potential to revolutionize the cosmetic photoprotective industry, only a few marketable products are currently based on liposomal nanostructured systems. This delay in market penetration can be attributed to obstacles such as the need for clinical studies, long-term stability assessments, industrial scale-up, and compliance with legal requirements for the commercialization of new nanocosmetics. Nevertheless, based on the comprehensive evidence presented in this work, leveraging liposomes as the foundation for new photoprotective products holds great promise for preventing cancer and other types of skin conditions caused by sun exposure.

## Figures and Tables

**Table 1:** Summary of studies demonstrating the use of liposomes, hybrid liposomes, and ethosomes in the development of photoprotective sun care products.

Formulations	Ultraviolet Filters	SPF Studies	Permeation Studies	References
Liposome of OMC and free OMC	Octyl methoxycinnamate	SPF in vitro of free OMC: 13.98 ± 0.66 SPF in vivo of free OMC: 7.00 ± 1.60 SPF in vitro of OMC-liposome: 13.88 ± 0.07 SPF in vivo of OMC-liposome: 11.50 ± 2.70 SPF in vivo of OMC-liposome after immersion: 5.80 ± 1.40	Tape stripping in vivo in humans: 22.64 ± 7.55 µg/cm^2^ of OMC-liposome in the SC after 240 min Tape stripping in vivo in humans: 14.57 ± 2.30 µg/cm^2^ of free OMC in the SC after 240 min	[65]
Free OMC, β-CD/OMC, lipo/OMC, and β-CD/OMC + lipo/OMC	Octyl methoxycinnamate	SPF in vivo of free OMC: 8.40 SPF in vivo after immersion of free OMC: 7.30 SPF in vitro of free OMC: 14.65 SPF in vivo of lipo/OMC: 11.00 ± 1.30 SPF in vivo after immersion of lipo/OMC: 10.30 ± 2.20 SPF in vitro of lipo/OMC: 15.05 SPF in vivo of β-CD/OMC: 8.50 SPF in vivo after immersion of β-CD/OMC: 6.50 SPF in vitro of β-CD/OMC: 14.80 SPF in vivo of β-CD/OMC + lipo/OMC: 11.60 ± 1.60 SPF in vivo after immersion of β-CD/OMC + lipo/OMC: 9.50 SPF in vitro of β-CD/OMC + lipo/OMC: 14.67	In vitro permeation in pig skin of free OMC: 11.95 ± 4.41 µg in epidermis and 12.38 ± 2.98 µg in dermis In vitro permeation in pig skin of lipo/OMC: 18.04 ± 1.17 µg in epidermis and 9.40 ± 2.36 µg in dermis In vitro permeation in pig skin of β-CD/OMC: 11.13 ± 3.36 µg in epidermis and 14.18 ± 3.59 µg in dermis In vitro permeation in pig skin of β-CD/OMC + lipo/OMC: 14.71 ± 2.39 µg in epidermis and 15.26 ± 3.47 µg in dermis	[74]
Liposomes of avobenzone and omega-3	Butyl methoxy dibenzoylmethane	Not reported	Not reported	[63]
Liposomes of CDBA	CDBA—derivative from butyl methoxy dibenzoylmethane	Not reported	Not reported	[75]
Liposomes of benzophenone-3	Benzophenone-3	Not reported	Not reported	[64]
Hybrid chitosan/liposomes of OMC, uncoated liposomes of OMC, free OMC	Octyl methoxycinnamate	SPF in vitro of free OMC: 7.30 ± 0.60 SPF in vitro of uncoated liposome with OMC: 8.00 ± 1.00 SPF in vitro of chitosan 0.1%/liposome with OMC: 9.70 ± 0.60 SPF in vitro of chitosan 0.30%/liposome with OMC: 9.70 ± 0.60 SPF in vitro of chitosan 0.50%/liposome with OMC: 10.30 ± 0.60	Not reported	[15]
Hybrid PHB/liposomes with *Coffea arabica* extract	Not reported	SPF in vitro of PHB/liposomes with roasted coffee extract: 50.45 ± 1.32 SPF in vitro of PHB/liposomes with green coffee extract: 37.65 ± 2.42 SPF in vitro of liposomes with green coffee and without PHB: 32.14 ± 0.64 SPF in vitro of roasted coffee extract: 47.89 ± 0.62 SPF in vitro of PHB/liposomes with roasted coffee extract after two months storage: 37.60 ± 0.30 SPF in vitro of PHB/liposomes with green coffee extract after two months storage: 27.50 ± 2.40 SPF in vitro of roasted coffee extract after two months storage: 1.90 ± 0.10	Not reported	[31]
Ethosome with naringin	TiO_2_ nanoparticles, ZnO nanoparticles	SPF in vitro of cream base: 0.71 ± 0.06 SPF in vitro of cream with naringin ethosomes and without ZnO and TiO_2_: 1.16 ± 0.09 SPF in vitro of cream with ZnO and TiO_2_, and no naringin ethosomes: 20.58 ± 0.58 SPF in vitro of cream with naringin ethosomes, ZnO and TiO_2_: 21.21 ± 0.62	In vitro skin permeation in excised rats’ skin of naringin suspension after 12 h: 306.81 ± 12.26 µg/cm^2^ permeated through the skin and 202.81 ± 9.45 µg/cm^2^ was retained in the skin In vitro skin permeation in excised rats’ skin of naringin ethosomes after 12 h: 325.38 ± 12.91 µg/cm^2^ permeated through the skin and 403.44 ± 15.33 µg/cm^2^ was retained in the skin In vitro skin permeation in excised rats’ skin of cream with naringin ethosomes and without ZnO and TiO_2_ after 12 h: 15.34 ± 0.61 µg/cm^2^ permeated through the skin and 13.35 ± 0.33 µg/cm^2^ was retained in the skin In vitro skin permeation of naringin in excised rats’ skin of cream with naringin ethosomes, ZnO, and TiO_2_ after 12 h: 14.91 ± 0.59 µg/cm^2^ permeated through the skin and 17.68 ± 0.42 µg/cm^2^ was retained in the skin In vivo skin permeation study of naringin of cream with naringin ethosomes, ZnO and TiO_2_ after 4 h: 550.20 ± 5.70 µg/cm^2^	[66]

β-CD: β-cyclodextrin; CDBA: 4-cholesterocarbonyl-4′0-(N,N0-diethylaminobutyloxy) azobenzene; lipo: liposome; OMC: octyl methoxycinnamate; PHB: polyhydroxybutyrate; SC: stratum corneum; SPF: sun protection factor; TiO_2_: titanium dioxide; ZnO: zinc oxide.

## Data Availability

No original data were reported (review article).
